# Current drugs for HIV-1: from challenges to potential in HIV/AIDS

**DOI:** 10.3389/fphar.2023.1294966

**Published:** 2023-10-26

**Authors:** Yuan Peng, Yanjun Zong, Dongfeng Wang, Junbing Chen, Zhe-Sheng Chen, Fujun Peng, Zhijun Liu

**Affiliations:** ^1^ School of Clinical Medicine, Weifang Medical University, Weifang, China; ^2^ Department of Medical Microbiology, School of Basic Medical Sciences, Weifang Medical University, Weifang, China; ^3^ School of Basic Medical Sciences, Weifang Medical University, Weifang, China; ^4^ Department of Liver Surgery and Transplantation, Zhongshan Hospital, Liver Cancer Institute, Fudan University, Shanghai, China; ^5^ Key Laboratory of Carcinogenesis and Cancer Invasion, Zhongshan Hospital, Fudan University, Shanghai, China; ^6^ Department of Pharmaceutical Sciences, College of Pharmacy and Health Sciences, St. John’s University, New York, NY, United States

**Keywords:** anti-HIV-1 drugs, monotherapy, combination therapy, HIV/AIDS treatment, predict HIV mutations, SERINC3, SERINC5

## Abstract

The human immunodeficiency virus (HIV) persists in latently infected CD4^+^T cells and integrates with the host genome until cell death. Acquired immunodeficiency syndrome (AIDS) is associated with HIV-1. Possibly, treating HIV/AIDS is an essential but challenging clinical goal. This review provides a detailed account of the types and mechanisms of monotherapy and combination therapy against HIV-1 and describes nanoparticle and hydrogel delivery systems. In particular, the recently developed capsid inhibitor (Lenacapavir) and the Ainuovirine/tenofovir disoproxil fumarate/lamivudine combination (ACC008) are described. It is interestingly to note that the lack of the multipass transmembrane proteins serine incorporator 3 (SERINC3) and the multipass transmembrane proteins serine incorporator 5 (SERINC5) may be one of the reasons for the enhanced infectivity of HIV-1. This discovery of SERINC3 and SERINC5 provides new ideas for HIV-1 medication development. Therefore, we believe that in treating AIDS, antiviral medications should be rationally selected for pre-exposure and post-exposure prophylaxis to avoid the emergence of drug resistance. Attention should be paid to the research and development of new drugs to predict HIV mutations as accurately as possible and to develop immune antibodies to provide multiple guarantees for the cure of AIDS.

## 1 Introduction

The human immunodeficiency virus (HIV), a positive RNA virus with a single strand, has two subtypes, HIV-1 and HIV-2. In 1981, HIV-1 was identified in young homosexual males. M (major), O (outlier), N (non-M, non-O), and the most recent group P, are the four groups identified. Group M viruses are further subdivided into nine subtypes (A-D, F-H, J, and K) (Bbosa et al., 2019). The HIV-1 subtype is associated with acquired immunodeficiency syndrome (AIDS) pathogenesis and progression. Total HIV-1 DNA level is a significant predictor of progression to AIDS. *In vivo*, higher HIV-1 DNA levels were detected in patients infected with the recombinant HIV-1 strain (CRF01_AE), which means more pathogenic (Lyu et al., 2020). Extending the scope of whole-genome sequencing to enable the correct classification of recombinant viruses is vital for AIDS diagnosis, anti-retroviral therapy, and the development of vaccines.

**FIGURE 1:**
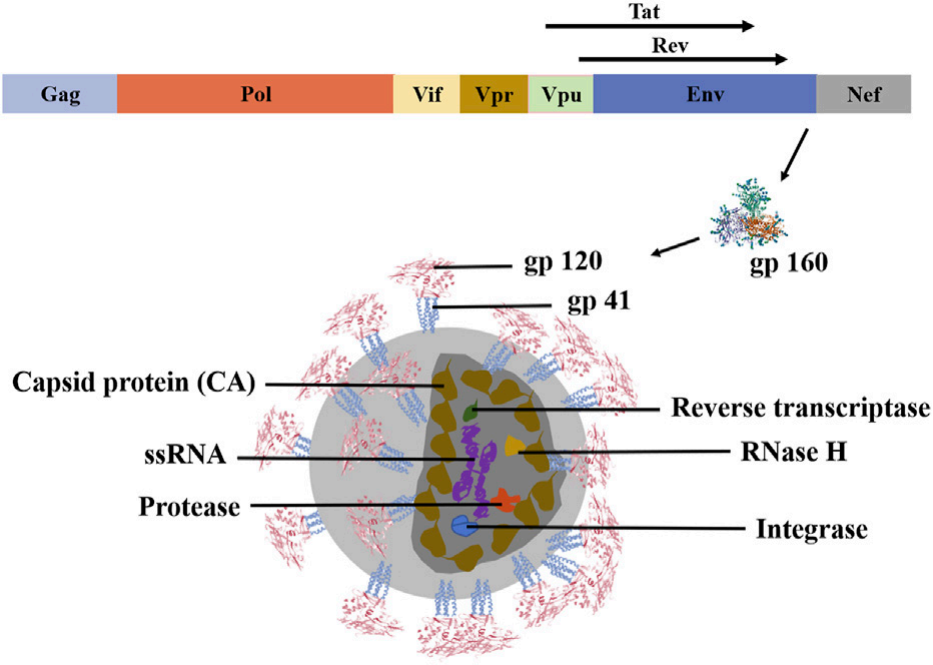
Molecular biological structure of HIV-1. HIV-1 is a single-stranded positive-stranded RNA virus with a genome containing structural genes (Gag, Pol, and Env) and regulatory genes (Vif, Vpr, Vpu, Nef, Tat, Rev, and Env). Among them, the regulatory gene Env transcribes and translates the gp160 protein. This protein is cleaved by the host protease into gp120 and gp41 to form the envelope glycoprotein spines. Reverse transcriptase, protease, integrase, and RNase H are scattered within the CA. These structures work together to make HIV-1 biologically active (Frankel and Young, 1998).

Three structural genes (Gag, Pol, and Env) and six regulatory genes comprise HIV-1 (Tat, Nef, Vif, Rev, Vpr, and Vpu). Env encodes gp160, which is intracellularly broken into gp120 and gp41 by the host protease (Frankel and Young, 1998). Gag proteins competitively recognize viral RNA (vRNA) packaging signals and form dimers with vRNA, which is degraded into pieces by virus proteases to produce capsid protein (CA) (shown in Figure 1) (Rein, 2019). HIV infects human CD4^+^T cells and damages the individual’s immune defense system. HIV-1 life cycle regulation depends on stage-specific HIV-1 gene expression. Specifically, Tat expressed early in viral infection accelerates the expression of viral genes. Simultaneously, Rev proteins play a crucial role in the transition from the early to the late stages of the HIV-1 life cycle, and they need to be degraded promptly after accomplishing their duty. Simultaneously, HIV-1 gene expression is influenced by the ubiquitin-proteasome system (UPS), which affects the HIV-1 life cycle (Rojas and Park, 2019). Initially, the viral surface protein gp120 attaches to the host cell surface receptor CD4. Under the influence of the C-C chemokine receptor type 5 (CCR5, R5) or CX-C chemokine receptor type 4 (CXCR4, X4), conformational changes lead to the exposure of gp41, which facilitates the fusion of the virus and host cell membranes. The virus then infects the host, releasing the genome for RNA replication (Rojas-Celis et al., 2019). Nef enhances HIV-1 infectivity by down-regulating the multipass transmembrane proteins serine incorporator 3 (SERINC3) and the multipass transmembrane proteins serine incorporator 5 (SERINC5) (Usami et al., 2015). Consequently, we expected that SERINC3 and SERINC5 would be crucial HIV diagnostic and therapeutic targets (Cano-Ortiz et al., 2023). In the early stages, some viruses reproduce explosively, triggering an immune response that reduces viral quantities in the blood. Unique to HIV infection, the virus reproduces continuously and chronically throughout time, transitioning from acute to chronic (Fauci et al., 1996). Moreover, HIV-1 viruses can be transferred through cell-to-cell contact, evading extracellular innate and adaptive immune systems (Pedro et al., 2019).

## 2 Single-ingredient drugs for anti-HIV

**FIGURE 2:**
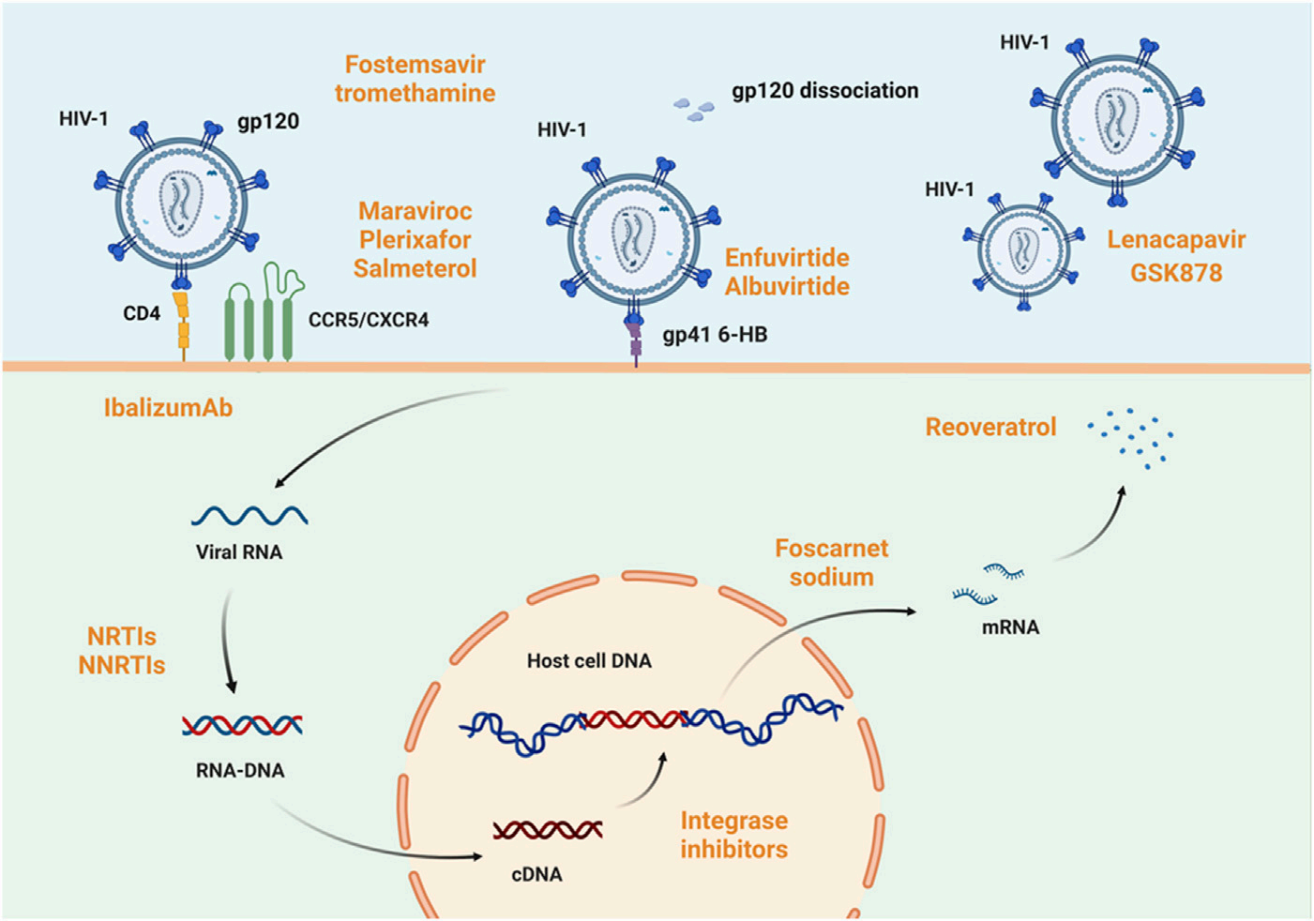
Host mechanism of HIV-1 infection and targets of anti-HIV-1 drugs. HIV infection of cells involves five processes: adsorption, invasion, decapitation, synthesis, and assembly. The viral glycoprotein spike gp120 binds to CD4 on the host cell surface. Under the mediation of CCR5 or CXCR4, the virus fuses with the host cell membrane. In this process, fostemsavir tromethamine (Wang et al., 2021) is active on gp120, enfuvirtide (Monteiro et al., 2021; Yeruva and Lee, 2022) and albuvirtide (Chong et al., 2012) act on gp41 6-HB, maraviroc, plerixafor, and salmeterol function on CCR5 or CXCR4 (Woollard and Kanmogne, 2015; Mirza et al., 2020; Wang et al., 2020), and ibalizumab (Bettiker et al., 2018) acts on CD4 receptor in host cells. All of these drugs’ act to prevent viral adsorption or invasion. When viral RNA is reverse-transcribed to form RNA-DNA and enters the host cell nucleus, integrase inhibitors such as cabotegravir sodium prevent its integration into host DNA. Even if viral genetic material is already successfully integrated, foscarnet sodium (Devianne-Garrigue et al., 1998) and resveratrol (Zhang et al., 2009) can prevent its transcription and translation to form viral proteins, respectively. Impressively, the capsid protein inhibitor lenacapavir and GSK878 are now available and efficiently blocks viral infection of host cells (Segal-Maurer et al., 2022). Created with BioRender.com.

The objective of treating AIDS at this stage is to suppress viral replication maximally in patients and enable them to rebuild and sustain immune function to combat non-AIDS-related disorders. In addition to antiviral medication, comprehensive AIDS treatment stresses supportive therapy and the restoration or improvement of immunological function. It also prevents consequences like cancer and opportunistic infections. According to the specific targets of antiviral medication action, antiviral medications are categorized as follows: drugs to block viral entry (including fusion inhibitors, CD4 adhesion inhibitors, and CCR5 inhibitors), antiretroviral medications (including nucleoside and non-nucleoside reverse transcriptase inhibitors), protease inhibitors, integrase inhibitors, and coat protein inhibitors. Highly active antiretroviral therapy (HAART), or ‘cocktail’ therapy (Lu et al., 2018), refers to the combination of two nucleoside reverse transcriptase inhibitors with a non-nucleoside reverse transcriptase inhibitor or protease inhibitor (shown in Figure 2). Moreover, gene editing has significantly affected HIV/AIDS prevention and treatment (Zhang et al., 2022).

### 2.1 Blocking viruses entry cells

Class I fusion protein Envelope glycoprotein (Env) is expressed on HIV surfaces and attacks CD4^+^T cells, macrophages, and monocytes. It enhances the attachment of viral particles to target cells and the fusion of viral and target cell membranes. It comprises three identical glycoprotein precursors, gp160, split into two subunits: the surface glycoprotein gp120 (SU) and the transmembrane structural domain gp41 (TM), during transit to the cell surface. The gp41 has a similar structure to the fusion-mediated component of all class I fusion proteins, with the fusion peptide located at the N-terminal end and the C-terminal end lodged in the virus membrane. During infection, the engagement of the gp120 receptor binding domain with the CD4 cell receptor initiates a sequence of conformational changes in the envelope protein. This triggers gp120 dissociating from gp41, exposing and refolding gp41, and promoting membrane fusion. Furthermore, chemokine receptors such as CCR5 and CXCR4 are required for viral entry. HIV entrance inhibitors are broadly categorized into three groups: fusion inhibitors targeting gp120 and gp41, CD4 adhesion inhibitors, and chemokine receptor inhibitors (Siegert et al., 2006).

#### 2.1.1 Fusion inhibitors

Enfuvirtide (ENF, T20) was the first HIV entrance inhibitor to authorization (Alaofi, 2022). Similar to the C-terminal repeat region (CHR) of gp41, it is called an analog, and blocks the interaction between the highly conserved region N-heptad repeat (NHR) and the gp41 CHR region in the pre-capillary intermediate (PHI), then prevents the synthesis of gp41 6-HB (CHR and NHR folding) (Monteiro et al., 2021). Residue E143 of the T20 peptide plays an essential role in the conformational structure of the peptide, and the E143A mutation affects not only the conformation of the T20 peptide but also its binding interactions with the HIV-1 receptor. This discovery has significant ramifications for optimizing and developing HIV-1 inhibitor peptides (Alaofi, 2022). Adonis Rubio et al. created D5-AR mutants based on monoclonal antibodies and the antiviral mechanism of ENF. These mutants enhance antiviral activity and support a vaccine strategy against PHI by eliciting antibodies against gp41 NHR (Rubio et al., 2021).

The conventional ENF target delivery system is susceptible to difficulties, such as a lack of specificity or inadequate medication delivery without the stimulus. To refine this technology, Taj Yeruva et al. constructed a peptide sequence as a stimulant reaction molecule directly attached to a polyethylene glycol (PEG)-based hydrogel matrix to permit proteolysis *via* enzyme stimulation. It protects women against HIV-1 infection (Yeruva and Lee, 2022). Tiago Figueira et al. postulated that a nanocarrier consisting of large unilamellar vesicles (LUVs) might be utilized to transport two HIV-1 inhibitors, ENF and protoporphyrin IX (PPIX), in combination. ENF and PPIX are, respectively, fusion and attachment inhibitors whose intrinsic lipophilicity results in their spontaneous incorporation into the lipid bilayer of the LUVs nanocarrier, hence boosting the synergistic action of the entering inhibitor (Figueira et al., 2020). Long-acting efavirenz-enfuvirtide co-loaded polymer-lipid hybrid nanoparticles (EFA-ENF-PLN) enabled a slow and sustained release of the two drugs from the polymer-lipid hybrid nanoparticles (PLN), and coumarin-6-loaded PLN contributed to the fight against the HIV-1 virus by increasing the uptake by T cells and macrophages (Surve et al., 2020).

Albuvirtide (ABT) is a 3-methyl imidazole (MPA)-modified HIV fusion inhibitor peptide that irreversibly binds to serum albumin and has a long half-life and potent anti-HIV activity *in vivo*. ABT can establish a stable α-helical conformation with the target sequence and inhibit, in a dominant-negative manner, the creation of a fused active hexa-helix bundle (6-HB), thus preventing HIV-1 Env-mediated membrane fusion and viral entry. ABT demonstrates potent inhibitory effects against HIV subtypes A, B9, and C, which dominate the global epidemic. It also inhibits the recombinant subtypes CRF07_BC and CRF01_AE. Moreover, ABT was particularly effective against HIV-1 strains resistant to T20 (Chong et al., 2012).

The gp120-binding inhibitor, fostemsavir, was approved in July 2020. As a novel HIV-1 inhibitor, it is particularly beneficial for regulating viral levels in HIV-1-infected persons with extensive therapy experience (Wang et al., 2021). Temesavir decreases gp120-mediated immunomodulatory activity, including the elimination of antibody-dependent cytotoxicity (ADCC) in uninfected bystander CD4^+^T cells, as well as gp120-induced cytokine bursts in peripheral blood mononuclear cells (PBMCs), implying that Fostemsavir may provide clinical benefits beyond blocking viral entry (Richard et al., 2023).

#### 2.1.2 Attachment of CD4 to inhibitors

Ibalizumab was the first monoclonal antibody approved for treating HIV-1 infection, and it played a significant role in HIV treatment (Blair, 2020). Rather than altering HIV gp120 function with domain 1, stereotactic interference prevents gp120 from attaching to CD4. It binds to CD4 receptor domain two and does not induce immunosuppression (Bettiker et al., 2018). It is a valuable and urgent therapeutic option for individuals with multimedication-resistant HIV-1 infection (Blair, 2020); however, it carries the risk of hepatitis B virus reactivation (Kaplan et al., 2021).

A unique class of HIV-1 entry inhibitors, called peptide triazoles (PTs), can be doubly combined with CD4 receptors and co-receptor binding sites of the gp120 subunit of Env. This binding triggered a significant conformational rearrangement of Env, which in turn inactivates Env and enhanced exposure to essential and conserved neutralizable regions of gp41, providing new ideas for vaccine development (Carter et al., 2023).

#### 2.1.3 Co-receptor inhibitors

HIV-1 should connect to the host cell’s surface and bind to CD4 to enter host cells. During this phase, the gp120 V3 loop is accessible, and HIV-1 interacts with synergistic receptors. Different cytophilic viruses determine whether they bind CCR5, CXCR4, or both, depending on differences in charge distribution and stereotaxis due to residual replacement (Tan et al., 2013). During an infection, R5-tropic viruses dominate. The gp120-gp41 complex undergoes enormous conformational changes, creating a trimeric hairpin structure that unites the viral envelope with the host cell membrane (Zhang et al., 2020).

CCR5 is a G-protein-coupled receptor (GPCR) that regulates natural killer cells (NK) and regulatory T cells (Treg). CCR5Δ32 is the most researched genetic variant of the CCR5 gene, influencing nonviral illnesses and HIV protection (Ellwanger et al., 2020). It has been demonstrated that CCR5 activation inhibits neuronal CREB and MAPK activity and axonal rejuvenation following neuronal damage. Without neuroinflammation, a direct connection between the gp120 V3 domain and CCR5 impairs synaptic plasticity and reduces memory (Riviere-Cazaux et al., 2022). Maraviroc (MVC) is a CCR5 antagonist that prevents the interaction between HIV-1 gp120 and CCR5 by binding specifically to the CCR5 receptor. MVC, the first CCR5 inhibitor approved, effectively inhibits HIV-1 entry into cells and is well endured (Woollard and Kanmogne, 2015). MVC was investigated as a potential topical pre-exposure prophylaxis (PrEP) for HIV prevention. MVC has been determined to be well endured orally and topically (McGowan et al., 2022). Plerixafor, the first CXCR4 antagonist, was licensed for non-Hodgkin and multiple myeloma in 2008. The mechanism of action of plerixafor may be related to the modulation of hematopoietic stem cells, blockade of X4 HIV-1 infection, increased circulating neutrophils, lymphocytes, and monocytes, decreased bone marrow-derived suppressor cells, and enhanced cytotoxic T-cell infiltration in tumors. However, it remains unproven whether plerixafor has an anti-HIV-1 impact (Wang et al., 2020). Salmeterol, a beta-2-adrenergic drug used to treat asthma, in combination with an anti-HIV-1 drug may cause patients to develop Cushing's syndrome. Thus, continued development and reuse of salmeterol in AIDS treatment is a potential strategy (Frias et al., 2016).

### 2.2 HIV-1 reverse transcriptase inhibitors

**FIGURE 3:**
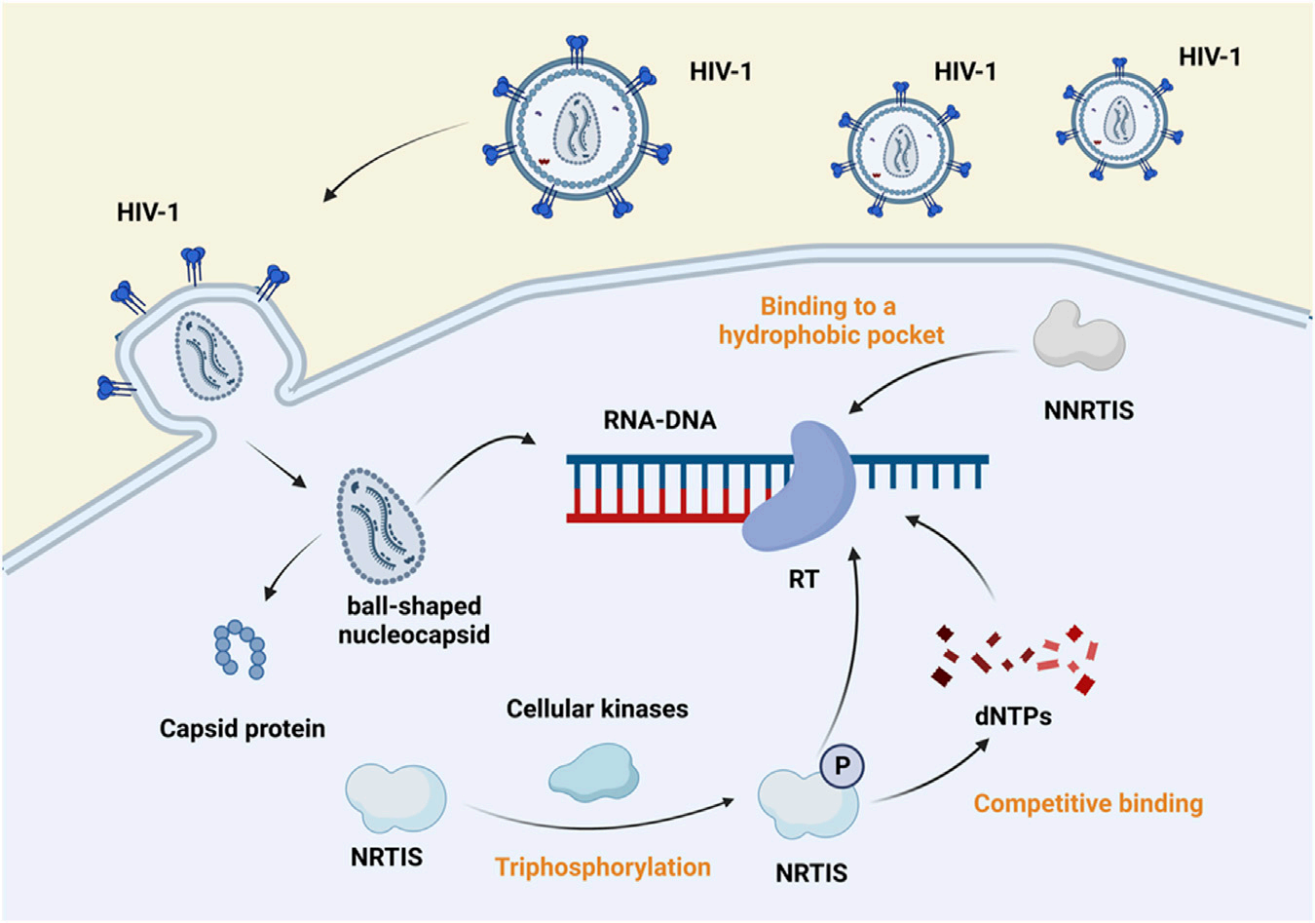
Mechanism of action of reverse transcriptase inhibitors. Reverse transcriptase inhibitors are classified into NRTIs and NNRTIs. The virus enters the host cell, sheds its capsid, and exposes genetic material. Cellular kinases phosphorylate the NRTIs and bind competitively with dNTP to template RNA, which blocks the transcription of viral genes. In contrast, NNRTIs bind to hydrophobic pockets close to the RT active site and inhibit viral gene transcription (Maga et al., 2010). Created with BioRender.com.

Inhibitors of HIV-1 reverse transcriptase are divided into nucleoside and non-nucleoside categories. Nucleoside reverse-transcriptase inhibitors (NRTIs), which compete with native nucleoside substrates and act as terminators of provirus DNA synthesis, and non-nucleoside reverse-transcriptase inhibitors (NNRTIs), which bind to hydrophobic pockets close to the site of reverse transcription activity, also perform an anti-retro translational function (shown in Figure 3) (Maga et al., 2010). In 1987, zidovudine (AZT), the first nucleoside reverse transcriptase inhibitor, was approved. Then followed didanosine, stavudine (d4T), lamivudine (3TC), and abacavir (ABC). In 2002, the drug adefovir dipivoxil (ADV) was licensed for oral administration. In advanced HIV disease, ADV is severely nephrotoxic and has no positive virological or immunological effects (Fisher et al., 2001). Despite therapy with ADV, HIV’s sensitivity to other medicines did not decrease (Miller et al., 2001). Emtricitabine (FTC) and tenofovir (PMPA/TFV) were approved in July 2003 and June 2008, respectively. Despite TFV efficacy in treating HIV-1 infection, there have been numerous reports of nephrotoxicity (Zhao et al., 2017). The approval dates for tenofovir disoproxil fumarate (TDF), tenofovir alafenamide (TAF) are October 2001 and November 2016. TAF is a predecessor to TFV with significant long-term effectiveness and tolerance (Moorthy et al., 2020). Physiologically based pharmacokinetic (PBPK) modeling provides a mechanistic way to assess drug biodistribution by constructing a particular PBPK model that matches rats’ treatment with gold nanoparticles (AuNP), such as stavudine-AuNP. It was further confirmed that AuNP can significantly increase the drug concentration in cell and tissue species (Zazo et al., 2022). ABC was converted to carbohydrate triphosphate, 3TC was transformed to lamivudine triphosphate, and AZT was intracellularly phosphorylated. These metabolites compete with deoxyguanosine triphosphate and deoxycytidine triphosphate to integrate into HIV DNA. They cease DNA replication and block reverse transcription, exerting antiviral effects (Yoshida et al., 2021).

Nevirapine (1996) was the first non-nucleoside reverse transcriptase inhibitor approved, followed by delavirdine (discontinued) (Wang et al., 2019). After that, efavirenz (EFV), etravirine (ETR), rilpivirine (RPV), and apricitabine were commercialized. Ainuovirine (ANV) was approved for commercial use in China in 2021, reducing the neurotoxicity of the first-generation non-nucleoside reverse transcriptase inhibitor, EFV, and overcoming the decrease in efficacy of the second-generation non-nucleoside reverse transcriptase inhibitor at higher viral loads (Su et al., 2023).

HIV-1-positive patients’ ABC hypersensitivity is closely related to HLA-B*57:01 gene carriage (Faridi et al., 2020). Except for AZT, most antiretroviral medications (ARVs) correct neutropenia. Concerns remain, however, regarding their impact on neutrophil function, namely the risk of persistent neutrophil activation. Neutrophil activation may predispose HIV-positive persons to chronic inflammatory disorders. The importance of platelets in the immune response to infection is becoming increasingly apparent. Even with antiretroviral medication, these cells contain HIV viruses and enhance viral transmission (ART). However, HIV-infected patients are typically characterized by thrombocytopenia, prolonged platelet activation, and dysfunction that may occur, exacerbating persistent immunological activation and an inflammatory vascular environment and increasing the risk of complications such as cardiovascular disease (CVD). Clinical trials have linked medications such as ABC and DRV to an increased CVD risk (Madzime et al., 2021).

Parents who are infected with HIV have the potential to transfer the infection to their offspring. The condition being referred to is commonly known as congenital infection. The use of ETR is generally well-tolerated and considered to be safe. The study found that children between the ages of 2 and 6 demonstrated higher viral effectiveness rates than children under the age of 2 (MacBrayne et al., 2021). Confirming ABC’s safety as a baseline medicine in neonates is still pending in HIV-1-positive individuals. While no adverse events were seen, a few neonates displayed symptoms consistent with anaphylaxis. In the context of drug administration to newborns, it has been observed that the utilization of ABC requires a reduction in the dosage of liquid ABC. However, additional researches are needed to evaluate the viability of employing solid formulations of neonatal ABC. These researches should specifically focus on assessing the feasibility of utilizing fixed-dose antiretroviral combinations (FDC), as these combinations have the potential to enhance the accessibility of comprehensive antiretroviral therapy (cART) for infants and children globally (Bekker et al., 2021). In order to overcome the constraints imposed by current anti-HIV medications, researchers are presently engaged in developing hydrophobic pharmaceuticals utilizing nanocarrier. Compared to conventional pharmaceuticals, it has been observed that anionic lipid vectors based on ETR exhibit much more efficacy in managing HIV-1 infection within HIV-infected cell lines. A notable improvement in the therapeutic index and pharmacokinetic properties accompanies this higher effectiveness. Therefore, utilizing a nanocarrier-based medication system with high hydrophobicity can serve as a targeted multi-site drug delivery system, offering therapeutic advantages in effectively eliminating HIV/AIDS infection (Rojekar et al., 2021).

### 2.3 Protease inhibitors

The HIV-1 protease consists of 99 amino acids that cleave the progenitors of the Gag and Gag-Pol polymers to produce mature, active proteins. The majority of HIV protease inhibitors are metabolized in the liver by the cytochrome P450 (CYP450) enzyme, which has a solid potential to inhibit the drug-metabolizing enzyme CYP450 3A4 (CYP3A4) (Dash and Boix, 2021), hence inhibiting HIV reproduction *in vivo* and attaining antiviral effects. Saquinavir, the first HIV protease inhibitor of the first generation, was launched in 1995 (Pereira and Vale, 2022). It was followed by ritonavir (RTV), indinavir (IDV), and nelfinavir (NFV). Amprenavir (APV) was also published in 1995, and fosamprenavir (FPV) is a promedication of APV (Arvieux and Tribut, 2005). Since then, lopinavir (LPV), atazanavir (ATV), tipranavir (TPV), and darunavir (DRV) have all been developed.

IDV was initially licensed as a autonomous drug. Physicians commonly prescribe RTV pharmacokinetic ‘boost’ in clinical practice to eliminate dietary restrictions, reduce pills, and make twice-daily dosing easier (Boyd, 2007). Liposomal nanoparticles (LNPs) specifically target CD4 and deliver IDV to CD4^+^-HIV host cells have been developed. Added polyethylene glycol to LNPs reduces peptide immunoprecipitation and boosts anti-HIV activity (Endsley and Ho, 2012). For pediatric, adolescent, adult, and gestational HIV infections, NFV is accessible. However, long-term drug resistance to NFV may result in mutations in HIV-1 D30N (Perry et al., 2005). In chemotherapy-resistant cancer cells, via endoplasmic reticulum/non-folding protein accentuating responses, NFV produces selective mitochondrial-independent cell death. In addition, NCV has antimicrobial properties against malaria, tuberculosis (TB), and SARS (Bruning et al., 2010), as well as echinococcosis (Liu et al., 2022), which has been demonstrated to ameliorate lung pathology generated by SARS-CoV-2 in animal models (Foo et al., 2022). DRV and LPV are suggested for resistance infections (Wong-Sam et al., 2022). ATV is an HIV protease inhibitor that decreases bilirubin binding competitively, and serum bilirubin levels are inversely related to cardiovascular risk. Compared to individuals receiving DRV, which did not elevate serum bilirubin, patients using ARV had a lower risk of cardiovascular disease and increased survival, which shows that creating a persistent rise in serum bilirubin could be a potential treatment strategy for reducing cardiovascular risk (Li et al., 2020). DRV combined with two NRTIs resulted in long-lasting viral suppression, even in patients with prior NRTI resistance. In addition, there is no risk of drug resistance, and a more prominent position in public health methods should be considered (Paton et al., 2021; Paton et al., 2022). TPV causes temporary and typically asymptomatic rises in blood aminotransferase levels, a rare cause of clinically apparent acute liver damage. During TPV therapy, patients with chronic hepatitis B or C exacerbations may experience liver injury (King and Acosta, 2006). The HIV-1 M46I mutation generated a structural kinetic shift that diminished the saquinavir effect without altering the protein’s activity. These findings could be exploited to build inhibitors of HIV-1 proteases resistant to durgs (Rana et al., 2022). Muscular tiredness, weakness, and pain caused by ATV, ERV, and RTV result from their disruption of the calcium homeostasis of the skeletal and cardiac sarcoplasmic reticulum (SR) muscles (Alomar et al., 2021). Computer analysis was performed on 14 HIV-1 proteinases (HPS/002, HPS/004, HPS/006, HPS/007, HPS/008, HPS/009, HPS/010, HPS/011, HPS/012, HPS/013, HPS/014, HPS/020, and HPS/024) to identify potential new antiviral drugs. The IC50/HIV-1 inhibition percentage, drug metabolism, and safety profiles of HPS/002 and HPS/004 indicate their suitability for clinical trials and research (Okafor et al., 2022).

### 2.4 Integrase inhibitors

**FIGURE 4:**
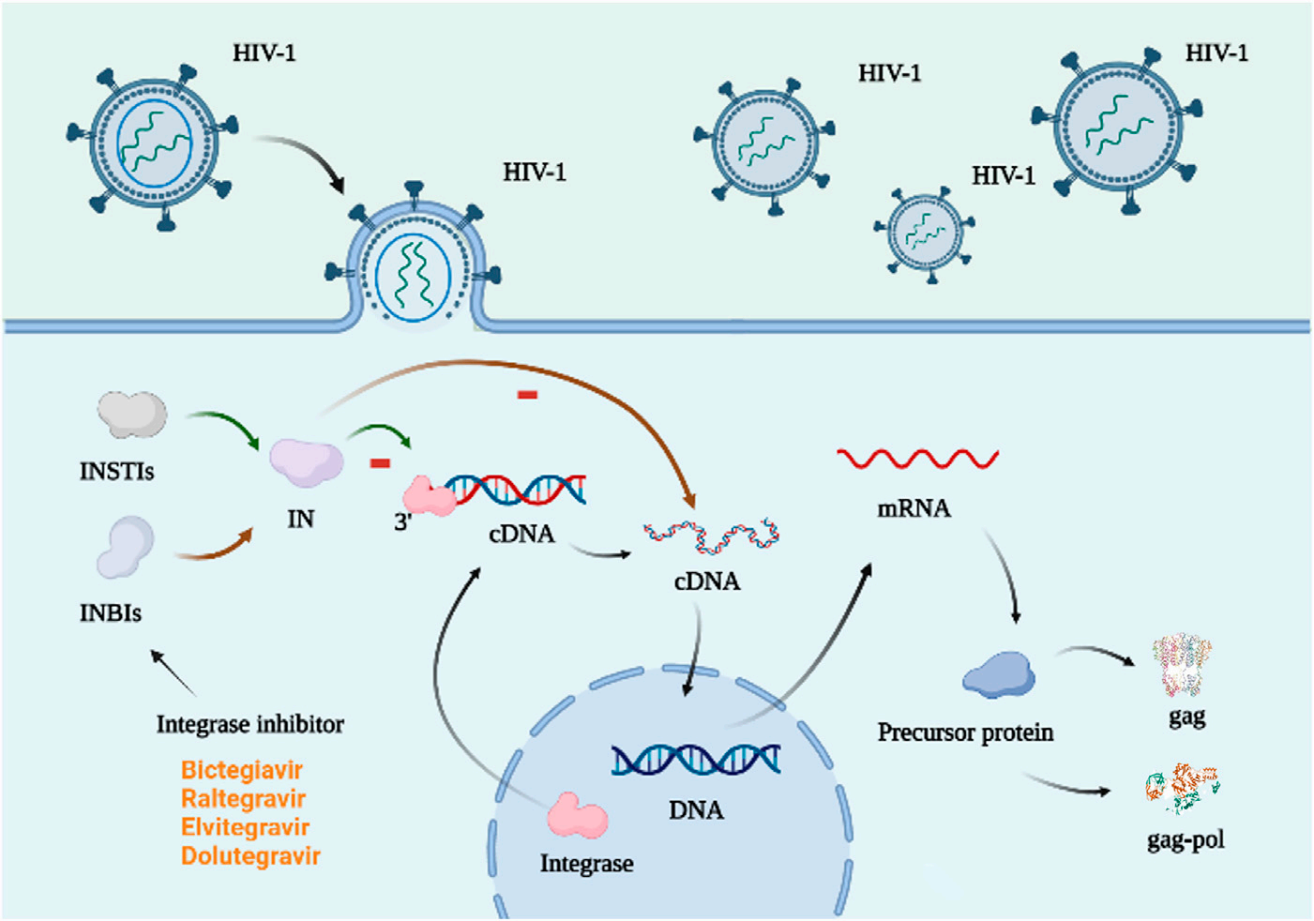
Mechanism of action of integrase inhibitors. The integrase is in the host cell and attaches to the 3′ end of the virus DNA, initiating the integration of the viral cDNA into the host DNA, which in turn is transcribed to form the precursor protein. This process requires a functionally intact integrase and the integrity of the last 10–20 base pairs at both ends of the viral cDNA (Aquaro et al., 2020). There are two types of integrase inhibitors: INSTIs and INBIs. The former attaches to the catalytic core domain of the integrase and prevents the enzyme from binding to DNA. The latter binds to the heterologous pocket of IN and hinders the conformational changes required for the strand transfer reaction (Trivedi et al., 2020). Created with BioRender.com.

Integrase (IN) is necessary for lentiviral replication (Jurado and Engelman, 2013), and integrase inhibitors assist HIV in integrating genetic information-carrying DNA into host DNA, which is typically carried by the virus but absent from host cells. Integrase inhibitors are divided into IN-chain-transfer inhibitors (INSTIs) and IN-binding inhibitors (INBIs). The former binds to the enzyme’s catalytic core domain, inhibiting its binding to dsDNA and the latter attaches to the IN’s foreign pocket, preventing conformational adjustments essential for a chain reaction (shown in Figure 4) (Trivedi et al., 2020). Common integrase inhibitors include raltegravir (RAL), elvitegravir (EVG), dolutegravir (DTG), and bictegravir (BIC) (Martin et al., 2021), all belonging to the INSTI class. Cabotegravir sodium (CAB), a member of the class of integrin-chain-transfer inhibitors (Engelman and Engelman, 2021), has shown remarkable safety in preventing HIV-1 in women in 2020 (Delany-Moretlwe et al., 2022). Long-acting cabotegravir injections (CAB LA) may be more effective during (PrEP) (Clement et al., 2020).

### 2.5 Viral capsid inhibitor

HIV-1 patients resistant to multiple antiretroviral drugs have few treatment alternatives. In August 2022, lenacapavir (Sunlenca) became commercially available (Mushtaq and Kazi, 2023). It is a weakly acidic indazole derivative with low water solubility and permeability. Lenacapavir selectively binds to the interface of the two hexameric subunits of the HIV-1 capsid, resulting in the development or inhibition of the immature HIV-1 capsid, interfering with capsid-mediated HIV-1 nuclear uptake, viral assembly and release, and capsid core formation during the HIV-1 life cycle (Dzinamarira et al., 2023). Furthermore, it demonstrated strong antiviral effectiveness in phase 1b research. Individuals with HIV-1 infection and multidrug resistance who received lenacapavir experienced a significant reduction in viral load compared to those who got a placebo at baseline (Segal-Maurer et al., 2022). Lenacapavir lacked cross-resistance to existing antiretroviral classes and was administered orally daily or weekly (Dvory-Sobol et al., 2022) or subcutaneously twice a year as an injectable solution (Paik, 2022).

GSK878 is a newly described HIV-1 inhibitor that binds to a mature capsid (CA) hexamer in a pocket identified initially as a binding site for the CA inhibitor PF-74 (Wang et al., 2023). GSK878 is involved in early (pre-integration) and late (post-integration) HIV-1 replication and is associated with altered stability in the HIV-1 CA core (Gillis et al., 2023).

### 2.6 Other antiviral drugs

Foscarnet sodium is tolerant to multiple viruses, including HIV, HSV, and HBV (Zeichner, 1998). As a polymerase inhibitor, it interferes with RNA synthesis and viral replication. Patients undergoing bisphosphonate drug demonstrated a decrease in plasma HIV-1 load (Devianne-Garrigue et al., 1998). In macrophages infected with antiretroviral medicines (AZT, EFV, IDV and ENF), type III interferons (IFNs) inhibit HIV-like viruses and improve anti-HIV action synergistically. IFNs can inhibit various strains, including the AZT-resistant virus (A012) and the reverse transcriptase inhibitor-resistant (TC49). Theoretically, IFNs can trigger multiple potent anti-HIV cytokines. These results strengthen the case that IFNs could be utilized to treat HIV infection (Wang et al., 2017). Moreover, resveratrol 3,4′,5-trihydroxydiphenylene may function as a potential HIV-1 therapeutic agent by reactivating SIRT1 closure induced by HIV infection and reducing the activity of the Tat protein of HIV, thereby preventing the activation of other HIV proteins and HIV spread to other cells. In addition, resveratrol inhibits several HIV cell enzymes in their reproduction. It also increases the development of enzymes necessary for activating nucleoside analogs. Therefore, resveratrol overcame past viral resistance to dihydroxy myosides (Zhang et al., 2009). Mupirocin, also known as pseudotumor A, is an isoleucine-tRNA synthetase that inhibits bacterial expression and is generated by fluorescent *pseudomonas*. It has been demonstrated to be efficient against various gram-positive and gram-negative bacteria (Mirza et al., 2020). As the only treatment proven to eradicate nasal obstruction in HIV-infected patients, *S. aureus* nasal carriage in HIV-infected patients was abolished after a few weeks (Martin et al., 1999), although the risk of relapse remained unchanged (Martin et al., 1999; Gordon et al., 2010).

## 3 Anti-HIV-1 combination drugs

### 3.1 Pharmacokinetic enhancer combination

Cobicistat (COBI) is an efficient inhibitor of the non-retroviral CYP3A4 enzyme, and when taken with antiretroviral drugs metabolized *via* the CYP3A4 route, it improves antiretroviral function. In addition to CYP3A4, COBI possesses inhibitory activity against CYP3A5, CYP2C9, CYP2D6, and the P-glycoprotein transporter (P-gp) (Dash and Boix, 2021). COBI is more acceptable, bioequivalent, and non-inferior to RTV (Deeks, 2014). It is non-inferior and bioequivalent to RTV and minimizes undesirable effects such as drug-drug interactions, fat degeneration, and hyperlipidemia. COBI has no anti-HIV action; hence, resistance to COBI as an add-on therapy is less of a concern (Putcharoen et al., 2015). Approved in August 2012, EVG/COBI/FTC/TDF (Stribild) is the first monotherapy regimen containing integrase inhibitors for HIV-1 infection (Olin et al., 2012). It consists of the integrated-chain-transfer inhibitor of HIV-1 (INSTI) elvitegravir, the pharmacokinetic enhancer cobicistat, and the nucleoside reverse transcriptase inhibitors emtricitabine and tenofovir (150 mg/150 mg/200 mg/300 mg) for once-daily treatment of HIV-1 infection in adults who have failed antiretroviral therapy. EVG/COBI/FTC/TDF is one of the preferred regimens (Perry, 2014) suggested for initial treatment and has a higher probability of achieving postexposure prophylaxis (PEP) than a multi-tablet regimen (MTR) (Malinverni et al., 2021). In November 2015, EVG/COBI/FTC/TAF (Genvoya) was approved and recommended as a first-line treatment. Regardless of the difficulty of employing cobicistat and other enhancers and the absence of a genetic barrier as high as with second-generation integrase inhibitors, EVG/COBI/FTC/TAF was a success in this population, with virtually no viral failure and no resistance mutations (Perez Stachowski et al., 2022). EVG/COBI/FTC/TAF is well tolerated, maintains HBV and HIV suppression, and improves proteinuria in HIV/HBV-coinfected individuals (Huang et al., 2021). Even the resistance mutation M184V/I did not affect the efficacy of E/EVG/COBI/FTC/TAF (Perez-Valero et al., 2021). In 2015, DRV/COBI (Rezolsta) became the first fixed-dose combination of protease inhibitors to be licensed for the treatment of HIV. Using DRV/COBI on healthy volunteers proved COBI’s efficacy and demonstrated the drug’s safety and efficacy in phase III research (Navarro and Curran, 2016). During pregnancy, increased CYP3A activity may contribute to the metabolism of DRV and COBI. Thus, regular DRV/COBI therapy during pregnancy may increase the risk of virological failure and perinatal transmission (Momper et al., 2021). However, it also suppresses viral load effectively (Dash and Boix, 2021.). ATV/COBI (Evotaz) was licensed for commercial usage in January 2015 and was efficacious and well tolerated in individuals with extensive HIV infection (Gallant et al., 2017). Randomized clinical trials demonstrated that ATV/c and ATV/RTV have equal efficacy and safety, long half-lives (Elliot et al., 2017), low rates of virological failure, and no ATV-resistant mutations. According to experts, ATV/COBI represents a new chance to expand the approach of switching to dual therapy to lower the danger of long-term harmful consequences (Antunes, 2017). The standard dosage of ATV/COBI during pregnancy may increase the likelihood of virological failure and perinatal transmission (Momper et al., 2022). COBI/DRV/FTC/TAF (Symtuza) 150/800/200/10 mg is the first single-pill daily regimen (STR) (Rashbaum et al., 2019) based on the protease inhibitor (PI) to treat HIV-1-infected adults and adolescents. It was introduced in September 2017. It combines the advantages of a genetic barrier to protease inhibition, pharmacokinetic enhancers, and nucleoside reverse transcriptase inhibitors (Deeks, 2018). COBI, DRV, FTC, and TAF revealed excellent renal and skeletal safety and CNS tolerance (Squillace et al., 2018).

### 3.2 Antiretroviral combinations

Using ABT-containing regimens (ABT/DTG or ABT/TDF/3TC) for HIV PEP demonstrates superior safety, tolerability, and adherence to non-ABT antiretroviral drug regimens (Nie et al., 2021). In addition, the novel dual-agent combination of ABT and LPV/RTV exhibited substantial dose-response correlations and could be evaluated in a broader patient group (Zhang et al., 2016). FTC is a member of the class of antiretroviral medicines that work when taken with TAF and TDF (Mayer et al., 2020). FTC/TDF and FTC/TAF were approved in 2004 and 2015. FTC/TAF increased renal safety compared to FTC/TDF but did not exhibit FTC/TDF lipid-lowering effects (Mesplede, 2021). Combined therapy with FTC and TDF effectively reduced HIV infection in male users, high-risk heterosexuals, and injectable drug users with shared devices. Drug resistance was infrequent but strongly associated with treatment duration and adherence. Combined therapy with FTC and TDF enhanced bone mineral density and kidney safety biomarkers. In addition, the concentrations of total cholesterol, high-density lipoprotein, and low-density lipoprotein dropped faster, consistent with their recognized lipid-lowering actions. Thus, the selection of preventive medicines before HIV exposure varies between populations (Ogbuagu et al., 2021; Fernando, 2018). FTC/RPV/TDF (Eviplera) was released in August 2011. Due to adverse effects involving the central nervous system and metabolic abnormalities, individuals were moved from an NNRTI-based regimen containing EFV, FTC, and TDF to Eviplera, and lipid distribution and CVR improved. Simultaneously, immunovirological control remained steady (Perez-Hernandez et al., 2014). The DTG/ABC/3TC triple therapy entered the market in August 2014 with everyday dosing, no augmentation, and a larger genetic resistance barrier (Christensen and Tan, 2022). ABC/DTG/3TC was launched in the same year. When switching regimens were evaluated for people with stable HIV-1 infection, switching to ABC/DTG/3TC was related to enhanced virologic effectiveness and safety (Trottier et al., 2017). DTG/RPV was introduced in November 2017 as the first dual antiretroviral STR (Dowers et al., 2018) to be used for maintenance therapy of HIV-1 infection. It was effective and safe for patients with viral suppression for at least 6 months (Sun et al., 2018). In February 2018, a single-agent regimen containing the integrase inhibitor BIC (Sax et al., 2021) was introduced as a secure and more practical option for patients already resistant to NRTIs (Stellbrink et al., 2020), without detectable emergent resistance or proximal tubular disease, and with improved gastrointestinal tolerability (Orkin et al., 2020b). Use during pregnancy was associated with the lowest composite of poor pregnancy outcomes and infant death (Lockman et al., 2021). AZT/3TC/NVP and EFV/3TC/TDF were introduced simultaneously.

Until June 2018, an EFV-based regimen was the optimal first-line treatment for HIV-1 infection, according to the World Health Organization (WHO). Because of side effects concerns, the combination of DTG and low-dose EVF was considered the first-line treatment for HIV-1 in areas with limited resources. The WHO suggested DTG in combination with two NRTIs for second-line therapy of HIV-1 infection (Calmy et al., 2020; Paton et al., 2021), and AZT replaced TFD as the first-line NRTI (Group et al., 2019). AZT is used with other anti-HIV drugs, including 3TC and ABC, to treat HIV infection. Effective in treating HIV-1-infected patients, particularly those with substantial NRTI resistance, is the combination of AZT, DTG, and NRTIs. As a second-line treatment, TDF is as effective as AZT. DOR/3TC/TDF was introduced in August 2018 and demonstrated no further resistance to EFV/FTC/TDF between weeks 48 and 96. It had fewer patients with sudden viral resistance and remained constant at week 96. Moreover, DOR/3TC/TDF showed improved safety, fewer neuropsychiatric side events, and a better lipid profile than the 2006-marketed EFV/FTC/TDF regimen (Orkin et al., 2021). In April 2019, DTG/3TC, a combination drug routinely used in clinical settings, was approved for commercial use. It efficiently maintained virological suppression (Llibre et al., 2023). It had a strong resistance gene barrier (Cahn et al., 2022), making it a viable first and subsequent therapy choice for HIV-1-infected individuals who are unknown or believed to be resistant to INSTIs or 3TC (Scott, 2020). CAB plus long-acting RPV, introduced in December 2019, can be used as an alternative to daily oral therapy for HIV-1-positive patients and may improve the ease of treatment, adherence, and quality of life for HIV-1-infected patients by reducing treatment frequency (up to six doses per year) (Overton et al., 2021). CAB/RPV therapy was non-inferior to oral DTG/ABC/3TC therapy in sustaining HIV-1 suppression, and patients reported improved satisfaction with treatment (Orkin et al., 2020a). BIC/FTC/TAF (Biktarvy) is approved for use in HIV-1 infection in both treatment-naive and treatment-experienced individuals after a series of successful phase III trials, and its clinical efficacy has also been confirmed (Peters and Iwuji, 2023). In March 2020, a CAB/RPV (Cabenuva) including a second-generation INSTI and NNRT was introduced, administered intramuscularly monthly (Howe et al., 2021), and viral failure was infrequent (Cutrell et al., 2021). In January 2023, ANV/TDF/3TC (ACC008) was approved, with a favorable safety profile (Huang et al., 2023).

## 4 Concluding remarks and future perspectives

To date, we have been able to better comprehend and analyze the genesis of DRM and its potential for transmission using next-generation sequencing (NGS) by evaluating HIV sequencing, drug susceptibility tests, and clinical data in a public database (Blassel et al., 2021). According to the literature, next-generation sequencing (NGS) techniques provide efficient methods by rapidly obtaining thousands to millions of short nucleotide sequences (Van Laethem et al., 2015). In addition, Sanger sequencing (SS) can be utilized to discover HIV resistance (DR), and pool-tagged pyrophosphate sequencing (TPP) can be used to identify low-abundance drug resistance variants at a reduced cost (Ji et al., 2013). Currently, most AIDS treatments are antiviral (inhibition of viral replication), and combination therapy is frequently employed to prevent drug resistance. HIV vaccine research has brought fresh insights into AIDS clinical prevention in recent years. RNA-based technologies are among the top candidates for gene therapies where they can be stably expressed for long-term HIV-1 suppression. Advances in gene and drug delivery strategies and improvements to non-coding RNA stability and antiviral properties will cooperatively drive progress in improving drug therapy and engineering HIV-1-resistant cells (Chen et al., 2023). The reported case series of HIV-cured patients in 2022 revealed that hematopoietic stem cell transplantation prevented HIV entrance into cells (Hsu et al., 2023). Thus, developing protective vaccines remains a top goal for HIV/AIDS epidemic control. Regardless of past immunogenic data, the ALVAC-gp120 regimen did not protect South African volunteers from HIV-1 infection (Gray et al., 2021). Although largely tolerated, immunization schedules at high mRNA dosages are challenging (Zhang et al., 2021). Based on RV144’s moderate efficiency, South African researchers launched a follow-up study with a modified vaccine design, the ALVAC-HIV vector, and the gp120 protein enhancer (Clade C strain) (Pitisuttithum and Marovich, 2020). A successful vaccination would considerably enhance and minimize health service strain. It is undeniable that vaccination has historically been the most cost-effective public health intervention required to eradicate infectious illnesses, with widespread diffusion and longevity (Dispinseri et al., 2022), and the problem of treatment resistance is still the key to healing for people with HIV-1. Thus, rational use of antiviral drugs, rational preexposure prophylaxis, and avoidance of the emergence of resistant mutations in the population and even the development of new resistant strains is essential. Using second-generation sequencing and other technologies to rationally predict the development of population resistance while attaching importance to vaccine development is critical to curing HIV-1-positive patients and preventing HIV infection.
